# Improvement course on evidence-based healthcare via teleconference

**DOI:** 10.1590/S1516-31802009000500014

**Published:** 2010-02-03

**Authors:** Cristiane Rufino de Macedo, Álvaro Nagib Atallah

**Affiliations:** I MD. Doctoral student, Universidade Federal de São Paulo - Escola Paulista de Medicina (Unifesp-EPM), São Paulo, Brazil.; II MD. Full professor and head of the Division of Emergency Medicine and Evidence-Based Medicine of Universidade Federal de São Paulo - Escola Paulista de Medicina (Unifesp-EPM). Director of the Brazilian Cochrane Center and Scientific Director of Associação Paulista de Medicina (APM), São Paulo, Brazil.

Evidence-based medicine came about in order to help healthcare professionals make better decisions regarding prevention, prognosis and treatment of the population as a whole.^1^ Today, medicine is not the only field utilizing the best available evidence; dentistry, physiotherapy and nursing, to name a few, are also doing so. In short, all fields of healthcare can be practiced using the best available evidence. Thus, the original concept of evidence-based medicine has been expanded to evidence-based healthcare.

These concepts have been introduced in different healthcare sectors in Brazil. Recently, the first improvement course on evidence-based healthcare was developed in Brazil for healthcare professionals within the network of Sentinel Hospitals. The goal of the course was to train healthcare professionals to make decisions based on the vast evidence available, thereby seeking to provide more benefits than risks to patients, and to promote conscientious and economic use of technological and financial resources made available by the Ministry of Health, when providing healthcare to the population.

The course was the result of a partnership between the Ministry of Health and the Teaching and Research Institute of the Hospital Sírio Libanês (Syrian-Lebanese Hospital, IEP-HSL) and was organized, coordinated and taught by Dr. Álvaro Nagib Atallah and the team from the Brazilian Cochrane Center. The content of the year-long course involved understanding the methodology for applying evidence-based healthcare. The 300 course hours were divided into weekly two-hour classes, year-round tutoring, weekly exercises and bimonthly evaluations (exams). The classes were taught on-line and were transmitted via live teleconference to a national network of 100 virtual classrooms in hospitals throughout Brazil.

Of the 2,000 students enrolled, 1,374 attended the course lectures and 957 submitted final projects in the form of structured, relevant research projects. All students received passing grades in the exams and handed in final projects to complete the course. The impact of this course was measured by means of personal, voluntary statements given by the students. We received more than 500 statements that indicated student satisfaction upon completion of the course. The following are two excerpts from the evaluation statements:

Statement 1: “The methodological focus on healthcare activities is fundamental. Without this, we would not be engaging in scientific activity but, rather, something close to witch-doctor practice. It is very disheartening to be among certain professionals whose actions are based on ‘I believe’ or ‘from my vast clinical experience.’ Evidence-based medicine, or rather healthcare, is an indispensable tool for those who wish to act professionally, ethically and scientifically. Thank you for the opportunity!”

Statement 2: “I feel better prepared to evaluate whether news or an article that appears in print has a scientific basis. I know where to search for scientific evidence.”

As a result of this experience, Brazil today has the largest evidence-based healthcare improvement course in the world,^2^ serving 3,000 healthcare professionals in public and private hospitals and healthcare clinics that practice rational use of medications. These initiatives surpass all the courses already offered in developed countries like the United Kingdom, Canada, Australia and others that regularly use evidence-based healthcare in their decision-making:^3^ countries that have already expressed an interest in the design and methodology used in this nationwide experience.

In the current year, about 3,500 health professionals are regularly attending the evidence-based healthcare course via teleconference.
